# Human Papillomavirus Across the Reproductive Lifespan: An Integrative Review of Fertility, Pregnancy Outcomes, and Fertility-Sparing Management

**DOI:** 10.3390/medicina61081499

**Published:** 2025-08-21

**Authors:** Matteo Terrinoni, Tullio Golia D’Augè, Giuseppe Mascellino, Federica Adinolfi, Michele Palisciano, Dario Rossetti, Gian Carlo Di Renzo, Andrea Giannini

**Affiliations:** 1 Department of Medicine and Surgery, University of Perugia, 06129 Perugia, Italy; 2 Department of Obstetrics and Gynecology, “Alto Tevere” Hospital of Città di Castello, USL Umbria 1, 06127 Perugia, Italy; dario.rossetti@uslumbria1.it; 3 Ospedale San Pietro Fatebenefratelli, 00189 Rome, Italy; tullio.goliadauge@uniroma1.it; 4 Dipartimento PROMISE del Policlinico di Palermo (Promozione della Salute Materno Infantile, di Medicina Interna e Specialistica G. d’Alessandro), 90127 Palermo, Italy; giuseppe.mascellino@unipa.it; 5 Department of Obstetrics and Gynecology, Azienda Ospedaliera di Perugia, 06129 Perugia, Italy; federica.adinolfi@ospedale.perugia.it (F.A.); michele.palisciano@live.it (M.P.); 6 Department of Obstetrics and Gynecology, “Branca” Hospital of Gubbio-Gualdo Tadino, USL Umbria 1, 06127 Perugia, Italy; 7 PREIS School (International and European School of Perinatal, Neonatal and Reproductive Medicine), 50121 Florence, Italy; profgcdr@gmail.com; 8 Department of Obstetrics, Gynecology and Perinatology, I.M. Sechenov First State University of Moscow, 119991 Moscow, Russia; 9 Unit of Gynecology, Sant’Andrea Hospital, Department of Surgical and Medical Sciences and Translational Medicine, Sapienza University of Rome, 00189 Rome, Italy

**Keywords:** human papillomavirus, fertility, pregnancy outcomes, vertical transmission, HPV vaccination, fertility-sparing surgery

## Abstract

*Background and Objectives*: Human papillomavirus (HPV) is the most prevalent sexually transmitted infection worldwide and, beyond its oncogenic potential, may impair reproductive health in both sexes. This review examines HPV’s effects on male and female fertility, obstetric outcomes, vertical transmission, and fertility-sparing management in oncology. *Materials and Methods*: A systematic search of PubMed, Embase, and Scopus was conducted using terms related to HPV and reproduction. Additional search terms included those related to therapeutic vaccines, antivirals, and genotype prevalence. English-language human studies reporting clinical reproductive outcomes were included. Thirty-seven studies met the inclusion criteria. Two reviewers independently screened and assessed study quality using a simplified GRADE framework. *Results*: In men, seminal HPV infection correlates with reduced progressive motility (SMD ≈ −0.85), abnormal morphology, and increased DNA fragmentation. In women, high-risk HPV doubles the odds of infertility (OR ≈ 2.3) and is associated with endometrial involvement. High first-trimester viral load predicts vertical transmission (aOR 6.4), which is also increased by vaginal delivery (RR 1.8) and is linked to PROM (OR 1.8) and preterm birth (OR 1.8). Modeling suggests that nine-valent vaccination plus 5-year HPV-based screening could reduce CIN2+ by up to 80% and excisional treatments by >75%. Fertility-sparing surgery in early cervical cancer yields a <4% recurrence and up to 68% live birth rates. *Conclusions*: This review uniquely synthesizes reproductive and oncologic impacts of HPV and emphasizes risk stratification, multidisciplinary prevention, and fertility preservation. Integration of HPV DNA quantification, personalized care, and vaccine-based strategies offers a path toward optimized outcomes in both sexes.

## 1. Introduction

Human papillomavirus (HPV) is the most widespread sexually transmitted infection worldwide, infecting nearly every sexually active individual at least once and accounting for approximately 5% of all cancers through its high-risk types [1,2]. HPV prevalence varies globally: pooled global prevalence among women is approximately 11.7%, with the highest rates in sub-Saharan Africa (>24%) and the lowest in Western Asia (<2%). The WHO’s 2020 strategy for cervical cancer elimination aims for 90% of girls to be fully vaccinated by age 15, 70% screening coverage, and 90% treatment of identified precancers by 2030. While Australia exceeds 80% vaccine coverage, parts of Eastern Europe and Central Asia remain below 30%.

Beyond its well-established oncogenic role in cervical cancer, HPV exerts multifaceted effects across the reproductive lifespan in both sexes [3,4]. In women, persistent infection leads not only to cervical dysplasia and cancer but also to an altered cervical microenvironment and impaired fertility that can compromise natural conception and assisted reproductive outcomes [5]. In men, recent systematic reviews have linked HPV to reduced sperm motility, abnormal morphology, increased DNA fragmentation and oxidative stress, all of which may decrease couple fertility and heighten the risk of pregnancy loss [6,7]. During pregnancy, HPV is capable of transplacental passage, with meta-analyses demonstrating significant associations with preterm delivery and premature rupture of membranes (PROM), as well as potential contributions to neonatal morbidity and mortality [8].

Given the scope and complexity of HPV’s impacts, including epidemiological patterns and molecular mechanisms of oncogenesis and sperm dysfunction to vertical transmission, clinical outcomes, and preventive strategies, our purpose is a clear rationale for a comprehensive, integrative review. Such a review should synthesize current evidence on HPV epidemiology, underlying biological pathways, fertility and gestational complications, vertical transmission dynamics, preventive interventions (including vaccination and barrier methods), and oncological management, with particular emphasis on conservative approaches to preserve fertility and reproductive health in young women [9].

While previous reviews have focused separately on HPV epidemiology, obstetric outcomes, or therapeutic approaches, we aim to integrate evidence regarding the effects of HPV on fertility in both males and females, the mechanisms and clinical outcomes of vertical transmission, the efficacy of preventive measures (vaccination, screening, and safe-sex practices), and current oncological management strategies with a clear focus on fertility-sparing interventions and multidisciplinary care across the reproductive lifespan.

We synthesize epidemiological data on HPV prevalence across age and geography, delve into the cellular and molecular mechanisms of HPV tropism and persistence in reproductive tissues, and critically appraise clinical outcomes in fertility, pregnancy, and neonatal health. We then review primary and secondary prevention and fertility-sparing treatments for HPV-associated neoplasms. Finally, we outline unresolved questions and propose a roadmap for future research. The manuscript is organized as follows: systematic search and appraisal methods, epidemiology, mechanistic insights, obstetric and neonatal outcomes, emerging therapies, fertility-sparing surgery, recommendations, highlighting gaps, and future directions.

## 2. Materials and Methods

### 2.1. Literature Search Strategy

We retrieved 3635 records from PubMed (1238), Embase (1415), and Scopus (982) for January 2000 to March 2025.

Search terms included “human papillomavirus” OR “HPV” in combination with “fertility,” “reproduction,” “pregnancy,” “neonatal,” “vaccine,” “screening,” and “therapy.” To capture emerging interventions and regional genotype data, supplementary searches focused on therapeutic HPV vaccines, antiviral agents, and geographic HPV genotype prevalence. We included English-language publications of human studies, including reviews and original research that reported clinical reproductive outcomes. We excluded non-human studies and any reports lacking clear clinical endpoints.

### 2.2. Study Selection

After removing 1526 duplicates, two authors independently screened 2109 titles and abstracts for relevance. Seventy-three full-text articles were assessed against prespecified criteria. Exclusion criteria were as follows:

- Non-human or in vitro studies;
- Lack of reproductive or obstetric outcome data;
- Lack of clear endpoints;
- Reviews, editorials, or conference abstracts without primary data;
- Case reports/series with fewer than 10 participants.

Thirty-seven studies fulfilled all inclusion criteria (four male fertility, six female fertility, eighteen obstetric outcomes, five vaccines, and four therapeutic vaccine trials; see Appendix A).

### 2.3. Data Extraction and Quality Appraisal

We extracted data on study design, sample size, HPV genotype(s), exposure and outcome measures, and effect estimates from each of the 37 studies. Study quality was rated using a simplified GRADE approach: RCTs were rated as high quality, cohort/case–control as moderate, and cross-sectional as low.

The use of GenAI has been reserved for image production.

## 3. Results

### 3.1. Effects of HPV on Fertility

#### 3.1.1. Male Fertility

Recent evidence highlights a clear association between seminal HPV infection and impaired sperm parameters in infertile men. A systematic review and meta-analysis of 10 studies (616 HPV-positive vs. 2029 HPV-negative infertile patients) demonstrated a significant reduction in progressive sperm motility among HPV-infected men (SMD −0.88; 95% CI −1.17 to −0.59) [10]. An SMD of −0.88 for sperm motility corresponds to a large effect, comparable in magnitude to that observed after varicocele repair (SMD ≈ −0.60) and antioxidant therapy (SMD ≈ −0.50) in subfertile men [11]. This suggests that HPV infection may contribute meaningfully to idiopathic male factor infertility and warrants consideration alongside other modifiable exposures in fertility workups. Similarly, a larger meta-analysis incorporating 25 studies (n = 6942) confirmed that HPV positivity correlates with decreased sperm motility (SMD −0.82; 95% CI −1.07 to −0.57) and abnormal morphology (SMD −0.52; 95% CI −0.84 to −0.21), while volume and concentration appear to be less affected [3,12].

High-risk HPV genotypes are particularly detrimental. In a cohort from North-Western Italy, HPV-51 and HPV-52 infections were each associated with a pronounced decline in sperm motility compared to uninfected controls, despite no differences in concentration or morphology overall [13]. Another study found that HR-HPV-positive men exhibited both lower progressive motility (*p* = 0.007) and higher sperm DNA fragmentation indices (SDFI; *p* = 0.003) than their HPV-negative counterparts, suggesting that viral presence may precipitate oxidative DNA damage [14].

##### Mechanisms of Impairment

Viral–Sperm Binding: HPV capsid proteins (notably L1) can adhere to the sperm head, potentially impairing membrane integrity and downstream motility functions.

Oxidative Stress and DNA Damage: Infection induces reactive oxygen species, elevating SDFI and promoting apoptotic pathways within spermatozoa.

While HPV-mediated oxidative stress has been described for multiple genotypes, emerging data indicate that high-risk types (particularly HPV 16 and 18) induce significantly greater reactive oxygen species (ROS) production than low-risk types (e.g., HPV 6, 11), likely reflecting differential E6/E7 interactions with mitochondrial and NADPH-oxidase pathways [3,14].

Immune–Mediated Effects: HPV may trigger antisperm antibody production, further compromising fertilizing potential and contributing to idiopathic infertility.

These findings underscore the importance of including HPV screening in the workup of idiopathic male infertility [15,16,17]. Given the documented impact on motility, morphology, and DNA integrity, HPV status should be considered when counselling couples and planning assisted reproductive techniques.

#### 3.1.2. Female Fertility

Persistent high-risk HPV infections in women have been linked to both cervical and endometrial alterations that may compromise natural conception and the success of assisted reproductive technologies (ART) [18,19]. A recent meta-analysis of 11 studies involving 15,450 women reported that high-risk HPV (HR-HPV) infection was significantly associated with female infertility (OR 2.33; 95% CI 1.42–3.83; *p* = 0.0008), whereas overall HPV (all genotypes) showed only a non-significant trend (OR 2.13; 95% CI 0.97–4.65; *p* = 0.06) [20].

Epidemiological data also suggest that HPV can ascend beyond the cervix—in a Brazilian cohort, HR-HPV DNA was detected in the endometrial cavity and peritoneal fluid of women with endometriosis at rates 5.4-fold higher than in controls (95% CI 1.07–97.25; *p* = 0.027), implicating HPV in upper genital tract pathology and possibly in infertility associated with endometriosis [21].

Mechanistic studies further support a direct role for HPV in early embryonic and implantation failure. Transfection of human blastocysts with the HPV-16 E6/E7 region induced a three- to six-fold increase in trophoblastic apoptosis and markedly reduced trophoblast invasion in vitro, effects that would predict early implantation loss even before clinical recognition of pregnancy [22].

When assessing ART outcomes, the evidence remains mixed but suggests modest impacts on embryo kinetics rather than on live birth rates. In a systematic review and meta-analysis of 10 studies (299 HPV-positive versus 2049 HPV-negative women), there was no significant difference in live birth/ongoing pregnancy (RR 1.16; 95% CI 0.88–1.53) or clinical pregnancy rates (RR 1.06; 95% CI 0.74–1.54), although subgroup analyses indicated poorer outcomes when the male partner was HPV-positive [23]. A more recent cohort study of women undergoing IVF found that HPV-positive patients had slightly altered embryo development kinetics (faster pronuclear fusion but slower progression to early blastocyst) yet achieved comparable live birth rates per started cycle (22.2% vs. 28.1%; *p*  >  0.05) [24].

Together, these data underscore that, while HPV infection in women does not uniformly preclude successful ART, it may contribute to implantation failures, subtle changes in embryo development, infertility, and, most of all, in the presence of endometrial involvement or concurrent male infection.

### 3.2. HPV in Pregnancy: Viral Load, Vertical Transmission, and Obstetric Outcomes

#### 3.2.1. Viral Load and Vertical Transmission

HPV viral load undergoes dynamic changes throughout gestation, generally showing a modest but statistically significant decline from the first to the third trimester (median difference −0.005 copies/cell; *p* < 0.05) [25]. Despite this overall decrease, elevated viral loads in the first trimester emerge as a strong predictor of mother-to-child transmission. In the multicenter Canadian HERITAGE cohort (n = 287 women infected with one of the 13 most common HPV genotypes), first-trimester viral loads of more than two copies per cell were associated with a more than sixfold increase in the odds of any HPV vertical transmission (adjusted OR 6.41; 95% CI 1.10–37.34) and an over 17-fold increase for HPV-16 specifically (adjusted OR 17.17; 95% CI 1.18–250.28) [26]. These findings held when viral load was analysed continuously or categorized using alternate cut-offs, underscoring its robustness as a biomarker.

In a separate prospective PLoS One study of 153 healthy pregnant women, HPV DNA was detected in 14% in the first trimester, 18% in the second, and 10% in the third. At birth, 5.2% of neonates were HPV-positive, and placental HPV DNA was identified in 3.3% of cases, all from mothers with at least one positive antenatal test [27]. Detection of HPV DNA in neonates correlated with maternal positivity in any trimester, reinforcing the concept that even transient or low-grade maternal infections can result in perinatal acquisition.

Meta-analytic data indicate that the overall rate of vertical HPV transmission ranges widely, from approximately 5% up to 72% with maternal viral load, lesion severity, and mode of delivery among key modifiers of risk [28]. Mechanistically, high maternal viral loads may facilitate transplacental passage via infected trophoblasts or ascend through microlesions in the cervical canal, seeding the placenta and fetal membranes. Detection of HPV DNA in cord blood, amniotic fluid, and neonatal mucosal samples further attests to multiple potential transmission routes [13,29].

Collectively, these studies establish maternal HPV viral load as a clinically meaningful biomarker for vertical transmission risk. Incorporating quantitative HPV testing into prenatal screening protocols could enable targeted monitoring of at-risk mother–infant dyads, prompt early pediatric follow-up, and inform discussions around delivery planning.

#### 3.2.2. Mode of Delivery and Obstetric Outcomes

The mode of delivery strongly influences both the risk of HPV vertical transmission and key obstetric outcomes:

Vertical Transmission Rates:

- A systematic quantitative review of nine cohort studies (2113 mother–infant pairs) found that infants delivered vaginally had a significantly higher risk of acquiring HPV DNA compared with those born by cesarean section (RR 1.8; 95% CI 1.3–2.4) [29].
- A later MDPI study of 432 dyads reported that only 3% of cesarean-delivered infants tested HPV-positive versus 18% of those delivered vaginally (adjusted OR 0.17; 95% CI 0.05–0.62), underscoring the birth canal as the principal route of perinatal exposure [30].

Premature Rupture of Membranes (PROM):

- In a Korean cohort of 311 women, high-risk HPV infection detected at six weeks postpartum was associated with a more than two-fold increased risk of PROM (adjusted OR 2.32; 95% CI 1.08–4.98) [31].
- A meta-analysis of seven studies (45,603 participants) confirmed that HPV-infected pregnant women had a higher probability of PROM (OR 1.74; 95% CI 1.45–2.10; *p* < 0.00001) [8].

Preterm Delivery:

- The same meta-analysis demonstrated that HPV infection increased the risk of preterm birth (OR 1.81; 95% CI 1.25–2.62; *p* = 0.002), suggesting that HPV-related cervical and membrane changes may precipitate early labour [8].
- Individual cohort studies have similarly linked vaginal delivery in HPV-positive women to higher preterm delivery rates compared with cesarean section, although the effect size varies with membrane rupture timing and lesion severity [30,32].

Low Birth Weight and Neonatal Morbidity:

- Several observational reports indicate that infants born to HPV-positive mothers via vaginal delivery are more likely to have low birth weight (<2500 g) and require neonatal intensive care, although pooled estimates remain limited by heterogeneity in study designs and outcome definitions [33].

In a large JAMA Network Open cohort of 899 pregnant women, persistent HPV-16/18 infection was associated with a markedly increased risk of all preterm births (adjusted odds ratio [aOR] 2.53; 95% CI 1.06–6.03) and spontaneous preterm births (aOR 2.92; 95% CI 1.09–7.81), after multivariable adjustment for maternal age, parity, and history of cervical intraepithelial neoplasia treatment [34]. In contrast, a population-based cohort of over 100,000 women in Australia found that treated Chlamydia trachomatis infection was not significantly associated with spontaneous preterm birth (aOR 1.08; 95% CI 0.91–1.28) once maternal smoking and socioeconomic status were included in the model [35]. The bacterial vaginosis was linked to a 41.7% increased risk of delivering a low-birth-weight preterm infant (41.7% vs. 19.0%, *p* = 0.010) independent of other recognized risk factors [36]. Finally, maternal smoking itself remains a potent confounder: in a PLoS Medicine “mega-cohort” analysis of over two million births, any prenatal smoking was associated with higher odds of preterm birth (e.g., aOR 1.17; 95% CI 1.16–1.19 for first-trimester smokers, rising to aOR 1.45; 95% CI 1.45–1.46 when smoking continued into the second trimester) [37]. Future studies should therefore employ harmonized screening for reproductive-tract co-infections and granular smoking exposure metrics (e.g., biomarkers, pack-years) to disentangle direct HPV effects from these overlapping pathways.

Collectively, these data implicate vaginal delivery in the presence of prolonged membrane rupture and high maternal viral load as a key risk factor for both vertical HPV transmission and adverse obstetric events such as PROM and preterm birth. While cesarean section appears to reduce transmission risk, its routine use solely for HPV prevention is not currently recommended without additional clinical indications [9,19,33,38,39,40].

#### 3.2.3. Long-Term Neonatal Outcomes

Emerging longitudinal cohorts have begun to characterize the persistence of HPV in infants and their early-life clinical trajectories:

- Persistence of HPV DNA: Several studies report that, despite clearance in many neonates, a notable minority retain detectable HPV beyond the perinatal period. In the Finnish Family Study, oral HPV carriage was detected in 14% of infants at birth and persisted in 10% by 26 months (mean persistence time 20.6 months; range 0.1–92.2 months), with α9-clade types (including HPV-16) most prone to long-term persistence. Similarly, a pooled analysis of smaller cohorts found that 10–25% of infants remained HPV-positive at 12 months, with persistence rates highest for HPV-6 and HPV-11 [41].
- Early Clearance versus Late Acquisition: The HERITAGE cohort (n = 1050 mother–infant pairs) showed 7% neonatal HPV positivity at birth but no persistence at 6 months, underscoring rapid clearance in most infants; however, late acquisition presumably through nonsexual close contact has been documented, suggesting ongoing risk beyond the perinatal window [25].
- Clinical Sequelae:
  - Juvenile-Onset Recurrent Respiratory Papillomatosis (JORRP): Though rare (estimated incidence 0.17–4.3 per 100,000 children), JORRP represents the most significant long-term morbidity of perinatal HPV transmission. Over 80% of cases are attributable to HPV-6 and HPV-11, both vaccine-preventable types. A U.S. registry study (1996–2002) confirmed these genotypes in nearly all pediatric papilloma biopsies and highlighted the protracted need for repeated surgical debulking and voice therapy [42].
  - Pulmonary Involvement: In a Beijing cohort of 192 children followed for a median of 10 years, 8.9% developed bronchial or pulmonary papillomatosis, a complication linked to earlier age of onset, higher frequency of interventions, and increased mortality risk (OR 94.9) [43].
- Neurodevelopment and Growth: To date, controlled neurodevelopmental assessments in cohorts up to 5 years old have not demonstrated major cognitive or motor deficits among HPV-exposed infants, although sample sizes remain underpowered for detecting subtle effects.

While the majority of newborns clear HPV rapidly, a meaningful fraction (10–25%) retain viral DNA into their first year, and a small subset develops significant morbidity such as JORRP or pulmonary papillomatosis. These findings underscore the need for extended follow-up to monitor for late-onset respiratory disease, growth deviations, and potential immunologic consequences. Ongoing surveillance in vaccinated populations will be particularly informative in determining the long-term protective effects of maternal immunization on neonatal and childhood HPV outcomes.

#### 3.2.4. Geographical Variation in Genotypes and Vaccine Uptake

Genotype Distributions

- Sub-Saharan Africa: Among invasive cervical cancer (ICC) cases, high-risk HPV types 16 and 18 together account for approximately 69.2% of infections (95% CI 66.0–72.4%), with HPV-35 (8.7%) and HPV-45 (7.4%) also frequently detected [44].
- Europe: In European ICC, HPV-16/18 prevalence is slightly higher (74% to 77% of cases) while in high-grade precancerous lesions (HSIL), these two types are found in about 52% of lesions (95% CI 50–54%) [45,46,47]. These regional differences in type distribution have implications for vaccine impact, especially where non-16/18 types (e.g., 31, 33, 52, 58) contribute more substantially to disease burden.

Vaccine Uptake

- Australia (>80% coverage): In 2023, 85.9% of Australian girls and 83.4% of boys had received at least one dose of the HPV vaccine by age 15. Australia’s school-based program with a single dose and a strong public-health messaging underpins these high rates [48].
- Eastern Europe (<30% coverage): Coverage remains below 30% in several Eastern European countries. For example, Armenia’s school-based program achieved just 23.7% coverage among girls aged ≤15 in early 2023 [49]. In Romania, only about 13% of eligible adolescents have ever been vaccinated, and fewer than 30% of women participate in routine cervical screening [50].
- Latin America (<30% coverage): Full-course vaccination coverage among females aged 10–20 years in Latin America and the Caribbean was estimated at 19.0% (95% CI 11.6–27.3) by 2014 [51]. Although some countries have since scaled up school-based delivery, regional averages still lag those in high-income settings.

HPV genotype prevalence and vaccine uptake vary markedly by region and resource context. In high-income countries, standardized screening and school-based vaccination achieve coverage rates > 80%. By contrast, in many sub-Saharan African and Latin American countries, coverage remains below 50% due to a constellation of barriers:

○ Structural: Limited cold-chain infrastructure compromises vaccine potency, and shortages of trained healthcare workers restrict outreach beyond urban centers.
○ Health system: Fragmented record-keeping leads to missed second-dose appointments, and national immunization programs often lack sustainable financing.
○ Sociocultural: Misinformation linking HPV vaccination with infertility, gender norms discouraging adolescent health visits, and mistrust of external donors all suppress demand.

Notably, community-led school-based campaigns in Rwanda and Bolivia have demonstrated the feasibility of over 70% coverage by coupling vaccination with menstrual hygiene education and door-to-door mobilization. Scaling such models—paired with mobile cold-chain units and electronic registries—may close the uptake gap in low-resource settings.

**Figure 1:**
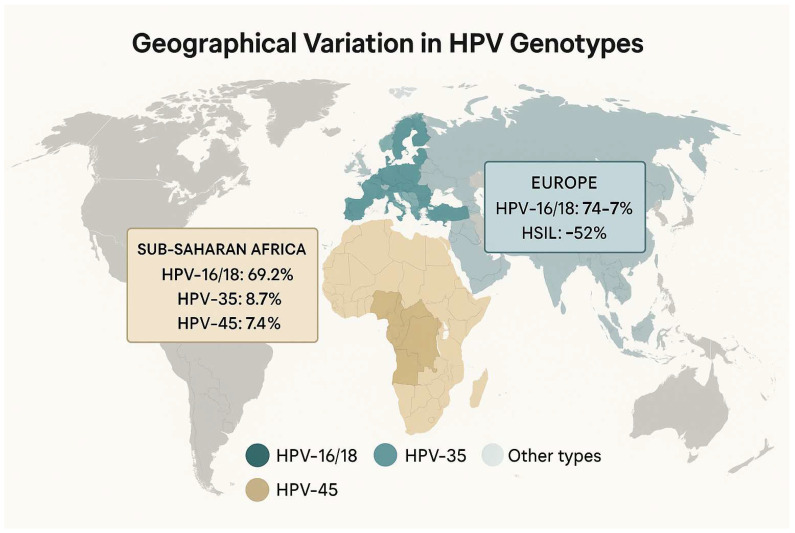
Geographical variation in HPV genotypes. “*Other types*”: all HPV genotypes outside of those listed (≥20 genotypes, most <5% prevalence each).

These stark disparities in both genotype prevalence and vaccine uptake are likely to translate into unequal risks of HPV acquisition and its reproductive sequelae. Tailoring vaccination strategies to local type distributions (e.g., using nonavalent vaccines where non-16/18 types are common) and strengthening a school-based or a community-driven delivery in underserved regions will be crucial to closing these gaps (Figure 1).

### 3.3. Vaccination and Screening: Preventive Implications

#### 3.3.1. Modeling the Preventive Impact

Mathematical models consistently demonstrate that the integration of high-coverage nine-valent HPV vaccination with extended-interval, HPV-based screening can dramatically reduce both disease burden and intervention-related reproductive harms:

- Reduction in Precancerous Lesions and Treatments:
  A BMC Medicine modeling study, assuming 85% uptake of the nine-valent vaccine with lifelong protection, projected lifetime reductions of 70–80% in CIN2+ and CIN3 cases compared with screening alone. When paired with primary HPV testing at 5-year intervals, the number of major excisional procedures was estimated to fall by over 75%, thereby averting a substantial proportion of treatment-related adverse obstetric outcomes such as preterm delivery and surgical complications [52,53].
- “Twice-Lifetime” Screening Strategy:
  In cohorts fully vaccinated with the nonavalent vaccine, simulation studies suggest that just two screens per lifetime, at ages 35 and 45, using primary HPV testing with cytology triage can preserve over 90% of the cancer-prevention benefits of more intensive screening, while reducing unwarranted colposcopies and excisional treatments by approximately 60–70% [54].
- Cost-Effectiveness and Gestational Outcomes:
  A U.S. model estimated that switching vaccinated women to HPV testing every 5 years (versus cytology every 3 years) yields incremental cost-effectiveness ratios below $50,000 per QALY and cuts the number of preterm births associated with excisional procedures by roughly 10–15% [55]. In the Netherlands, updated long-term vaccine effectiveness data incorporated into a cost-effectiveness framework predicted an 80% reduction in excisional treatments and corresponding averted preterm deliveries—translating into significant QALY gains and health-care savings [53,54,55,56].

These modeling insights underscore that, in vaccinated populations, scaling back screening frequency and targeting HPV testing can maintain high levels of cancer prevention while substantially lowering the need for invasive precancer treatments and improving also gestational outcomes.

#### 3.3.2. Indirect Benefits

By preventing HPV-related precancerous cervical lesions, vaccination and HPV-based screening indirectly improve reproductive outcomes by reducing the need for invasive excisional procedures, which are themselves risk factors for adverse obstetric events.

- Reduced Excisional Treatments and Preterm Birth:
  Meta-analyses of observational studies have established that any excisional treatment for cervical intraepithelial neoplasia (CIN) (LLETZ, LEEP, Cold knife conization, and laser conization) increases the risk of subsequent preterm birth (PTB) (RR ~1.75; 95% CI 1.57–1.96) compared to untreated colposcopy referrals. More radical excisional techniques confer even higher PTB risks (CKC: OR 2.27; 95% CI 1.70–3.02), whereas ablative methods such as laser ablation and cryotherapy do not significantly elevate PTB risk (laser ablation: OR 1.05; 95% CI 0.78–1.41). By reducing CIN incidence by up to 80% with high-coverage nine-valent vaccination, models predict an over 75% decline in excisional procedures with a commensurate drop in PTB rates and associated neonatal morbidity [57].
- Population-Level Reductions in Adverse Pregnancy Outcomes:
  Ecological analyses linking national HPV vaccination coverage to birth registries in Australia demonstrated that every 20% increase in three-dose vaccination coverage corresponded to a 1% reduction in preterm births and a 2% reduction in small-for-gestational-age (SGA) infants between 2000 and 2015 (adjusted for maternal age and birth year). Extrapolated, Australia’s HPV program may have prevented over 2000 preterm births and nearly 3000 SGA cases with a direct reproductive health benefit beyond cancer prevention [58,59].
- Economic Impact of Avoided Obstetric Complications:
  A budget impact analysis in Italy evaluated the vaccination of women post-CIN treatment with the nine-valent vaccine. By averting future excisional treatments and the roughly 10–15% of preterm deliveries attributable to those procedures, the strategy projected savings of EUR 155,596 over five years for the national health service, illustrating how indirect obstetric benefits translate into cost savings [60].

Collectively, these data underscore that the benefits of HPV prevention extend well beyond oncologic endpoints. By averting the need for cervical excisions and reducing preterm birth, low birth weight, and their lifelong sequelae, HPV vaccination and optimized screening protocols foster healthier pregnancies and neonates, amplifying the overall public-health impact of HPV control strategies.

### 3.4. Emerging Therapeutic Approaches

#### 3.4.1. Therapeutic Vaccines

Therapeutic vaccines targeting the HPV E6 and E7 oncoproteins aim to induce robust cell-mediated immunity to clear existing lesions while preserving cervical integrity and fertility:

- VGX-3100 (Inovio Pharmaceuticals): In a randomized, double-blind, placebo-controlled Phase IIb trial (ITT n = 167), three doses of VGX-3100 delivered intramuscularly with electroporation induced histopathological regression (to CIN 1 or normal) in 49.5% of women with CIN 2/3 (vs. 30.6% placebo; *p* = 0.034), and combined lesion regression with viral clearance in 40.2% (vs. 14.3%; *p* = 0.003) at 36 weeks post-treatment. Long-term follow-up demonstrated durable responses: among VGX-3100 responders who avoided excision, 91% remained HPV-16/18 DNA–negative and free of HSIL recurrence at 18 months [61].
- GX-188E (Genexine): This DNA vaccine encodes HPV16/18 E6/E7 fused to a tissue plasminogen activator signal and is currently in Phase II trials, both as monotherapy and combined with pembrolizumab, showing objective responses in up to 41% of advanced cervical cancer cases [62].
- VB10.16 (Nykode Therapeutics): A DNA-based vaccine targeting E6/E7 with a next-generation plasmid backbone; early Phase I data report favorable safety and immunogenicity, with lesion regression in approximately 30–40% of CIN 2/3 patients [63].
- RNA-Lipoplex Vaccines (e.g., BNT113): Leveraging mRNA encapsulated in lipid nanoparticles, BNT113 induces potent CD4^+^ and CD8^+^ T-cell responses against E6/E7, with ongoing Phase II studies combining it with checkpoint inhibitors in HPV16^+^ head and neck and cervical cancers [64].

These therapeutic vaccines offer a promising fertility-sparing alternative to excisional surgery, particularly for women planning future pregnancies.

#### 3.4.2. Antiviral Agents

Although no HPV-specific antivirals have yet advanced to late-stage clinical trials, several preclinical strategies are under investigation:

- Capsid Assembly Inhibitors: Inspired by hepatitis B virus capsid assembly modulators, novel small molecules designed to disrupt HPV L1 capsid formation have demonstrated in vitro inhibition of virion assembly and infectivity. However, none have entered human trials to date [65].
- RNA Interference (siRNA/shRNA): Multiple studies have employed siRNAs targeting HPV E6 and/or E7 transcripts in cervical cancer cell lines (e.g., SiHa, CaSki, HeLa), resulting in p53 accumulation, cell cycle arrest, and apoptosis. In murine xenograft models, intratumoral or systemic delivery of E6/E7-specific siRNAs produced significant tumor regression without overt toxicity [66].
- Topical and Intralesional Agents: Compounds such as cidofovir gel and intralesional interferon-α have shown modest efficacy in clearing anogenital warts and CIN lesions, but reproductive safety profiles remain insufficiently characterized [67].

#### 3.4.3. Limitations and Implementation Challenges

Despite promising immunogenicity and early efficacy data for DNA- and RNA-based HPV vaccines, several hurdles may limit real-world impact:

- Manufacturing and Distribution: Novel mRNA platforms require stringent cold-chain conditions (<−20 °C), creating logistical bottlenecks in regions lacking ultralow-temperature freezers.
- Cost and Affordability: Estimated production costs (USD $30–50 per dose) exceed those of conventional VLP vaccines, and comprehensive health-economic analyses remain sparse.
- Reproductive Safety: To date, no dedicated fertility-endpoint trials have been conducted; teratogenicity has only been assessed in small animal models, leaving human reproductive risks largely uncharacterized.

Addressing these gaps will require collaborative initiatives to fund fertility-safety studies, invest in decentralized cold-chain innovations, and negotiate tiered pricing agreements for low- and middle-income countries.

### 3.5. Management of Cervical Cancer in Young Women: A Conservative Approach

#### 3.5.1. Importance of Conservative Surgery

Fertility-sparing surgery (FSS) has become an essential strategy for young women with early-stage cervical cancer who wish to preserve childbearing potential. FSS encompasses a spectrum of techniques: non-radical procedures such as cervical conization and simple trachelectomy, as well as more radical approaches including vaginal, abdominal, and minimally invasive radical trachelectomy (RT).

- Oncologic Safety:
  - A comprehensive systematic review of 65 studies (3044 patients) reported a pooled cancer recurrence rate of 3.2% and a disease-specific mortality of 0.6% at a median follow-up of 39.7 months, with no significant differences across FSS modalities [68].
  - Non-radical procedures in selected low-risk patients (lesions < 2 cm, negative nodes) yielded even lower relapse rates: across 203 cases of conization or simple trachelectomy, the crude recurrence rate was 2.7% and mortality 0.5% [69].
  - In a large retrospective cohort of 733 women undergoing FSS, recurrence was observed in 7% and cervical-cancer-specific death in 2.6% after a median 72-month follow-up [70].
- Reproductive Outcomes:
  - Among women attempting conception after FSS, the mean clinical pregnancy rate was 53.2%, with the highest rate following vaginal RT (67.5%). The overall live birth rate post-FSS averaged 67.8%, and approximately 21% of conceptions required assisted reproductive technologies.
  - In the subset treated by non-radical surgery, 68% of successful FSS patients achieved live birth (71 live births among 124 women). These important findings must be analyzed considering the heterogeneity of the surgical techniques (cervical conization, simple trachelectomy) and that patients eligible for conservative therapy have more favorable prognostic factors that could also have influenced the live birth rate.
  - For tumors ≥ 2 cm managed with neoadjuvant chemotherapy (NACT) followed by less radical FSS, pregnancy rates approached 44% and live birth rates 45.5%, albeit with higher preterm birth (43.9%) compared to surgery-only cohorts [71,72].
- Procedure Selection and Outcomes:
  - Vaginal RT offers the highest pregnancy likelihood but requires expertise and careful patient selection; abdominal and minimally invasive RT provide comparable oncologic safety, with some variation in fertility metrics.
  - Conization or simple trachelectomy is appropriate for very early disease (stage IA1 without lymph vascular invasion), minimizing cervical trauma and the subsequent risk of obstetric complications [71].

Together, these data affirm that, in appropriately selected patients, conservative surgical approaches can achieve oncologic outcomes akin to standard radical hysterectomy while maintaining substantial fertility potential. Detailed preoperative counselling and multidisciplinary management are paramount to balancing cancer control with reproductive goals.

#### 3.5.2. Clinical Implications

Early diagnosis of HPV-related cervical pathology, combined with evidence-based screening protocols, is pivotal for identifying patients suitable for conservative, fertility-sparing interventions. Primary HPV testing nowadays is recommended as the preferred modality for women aged ≥25, enabling the detection of high-risk infections before the development of significant CIN, facilitating timely referral to colposcopy and eligibility assessment for fertility-sparing surgery (FSS) or ablative treatments [73]. Integrating HPV vaccination status into screening algorithms further refines risk stratification: vaccinated cohorts may safely undergo extended-interval, HPV-based screening (e.g., every 5 years) with minimal compromise in cancer prevention and reduced harms from over-treatment [55].

Multidisciplinary evaluation (gynecologic oncology, reproductive endocrinology, and maternal–fetal medicine) is essential for tailoring management. Prognostic models and nomograms that incorporate cone-length, time since conization, cervical length in the second trimester, maternal age, and obstetric history can accurately predict risks of preterm delivery and PROM following excisional procedures. For example, a validated nomogram demonstrated excellent discrimination (AUC 0.87) for predicting PROM and preterm birth in women post-conization, identifying those who may benefit from closer surveillance or prophylactic cerclage. Likewise, cervical length–based triage models have been shown to safely reduce unnecessary follow-up scans and optimize cerclage timing and suture selection, lowering preterm birth rates in this high-risk group [74,75].

Nine-valent Vaccine and Miscarriage Risk:

Recent safety data provide reassurance regarding inadvertent 9-valent HPV vaccine (9vHPV) exposure during or around pregnancy. In a cohort of 1493 pregnancies, exposures to the 9vHPV vaccine during gestation or peri pregnancy were not associated with an increased risk of spontaneous abortion (adjusted HR 1.12; 95% CI 0.66–1.93), preterm birth (RR 0.73; 95% CI 0.44–1.20), small-for-gestational-age birth (RR 1.31; 95% CI 0.78–2.20), or major structural birth defects. Conversely, a meta-analysis of peri-conceptional exposures, based on a single study, reported a higher spontaneous abortion rate when conception occurred within 30 days of 9vHPV vaccination (RR 2.04; 95% CI 1.28–3.24), underscoring the need for further research in larger cohorts [76,77].

### 3.6. Prognostic Models in Post-Conization Women

Accurate risk stratification for preterm premature rupture of membranes (PROM) and preterm birth after cervical conization enables tailored prenatal management like intensified surveillance, prophylactic cerclage, or progesterone therapy, in women at the highest risk.

- Xiu et al. Nomogram (2024) [74]:
  - Population and Design: Retrospective cohort of 100 women who conceived after cervical conization (2014–2023).
  - Independent Predictors:
    - Pre-pregnancy obesity (BMI ≥ 30 kg/m^2^)
    - Advanced maternal age (≥35 years)
    - Short conization-to-pregnancy interval (<12 months)
    - Second-trimester cervical length < 25 mm
  - Model Performance:
    - Discrimination: AUC 0.8746 (95% CI 0.815–0.935)
    - Calibration: Excellent agreement in both internal and bootstrap validation
    - Clinical Utility: Decision curve analysis demonstrated net benefit for threshold probabilities of 20–60%.
  - Clinical Application: The nomogram provides individualized PROM/preterm-delivery risk estimates to guide preconception counselling and mid-trimester interventions [78].
- Cone Volume and Healing Interval:
  - Leiman et al. (1980) found that larger excised cone volumes (>4 mL) were independently associated with increased preterm birth risk (OR ~1.8) and recommended careful volume reduction in young women desiring fertility [79].
  - Himes and Simhan (2007) demonstrated that a conization-to-pregnancy interval shorter than 3 months doubled the risk of preterm delivery compared with intervals ≥ 6 months, underscoring the need for adequate cervical healing prior to conception [80].
- Other Predictive Models:
  - A BMC Pregnancy and Childbirth model (2024) in low-risk women with mid-trimester short cervix (<25 mm) identified multiparity, leucocytosis, and cervical length as predictors of spontaneous preterm birth < 32 weeks (AUC 0.815), demonstrating the value of integrating inflammatory markers with ultrasonographic parameters [78].

These prognostic tools offer practical, validated means to quantify individual preterm-delivery risk post-conization. Their application supports personalized prenatal care, optimizing timing for interventions (e.g., cerclage, vaginal progesterone) and improving perinatal outcomes in this high-risk population.

### 3.7. Quantitative Summary of Key Outcomes

These aggregated estimates underscore the clinical importance of HPV not only as an oncogenic virus but also as a contributor to adverse obstetric and early-life outcomes. Their moderate to low GRADE ratings reflect the need for further high-quality, prospective research like randomized interventions to mitigate these risks (Table 1).

### 3.8. Risk-of-Bias Appraisal

A formal appraisal of study quality reveals:

- Randomized Controlled Trials (RCTs) of Prophylactic Vaccines:

Trials evaluating quadrivalent and nonavalent HPV vaccines uniformly demonstrate low risk of bias across domains of random sequence generation, allocation concealment, blinding, and outcome reporting. High retention rates and consistent case definitions further bolster confidence in vaccine efficacy and safety findings.

- Cohort Studies on Obstetric Outcomes:

These studies often suffer from moderate selection bias, as HPV testing is typically performed in tertiary-care settings or referral centers, limiting generalizability. Confounding by maternal age, smoking, socioeconomic status, and co-infections is inconsistently addressed, and loss to follow-up in longitudinal designs can skew estimates of vertical transmission and neonatal persistence. The overall risk of bias in this group is therefore judged as moderate [81].

- Cross-Sectional Microbiome and Biomarker Analyses:

Single-timepoint assessments of the cervical microbiome or viral load provide important hypotheses but are inherently limited by low quality of evidence: absence of temporality, potential measurement error, and small sample sizes. Without prospective validation, these cross-sectional findings remain exploratory and hypothesis-generating.

Overall Assessment:

While the RCT evidence for prophylactic vaccines is robust, translating into strong recommendations for broad immunization, observational studies on fertility and perinatal risks require cautious interpretation. Future research should prioritize well-designed prospective cohorts with standardized confounder adjustment and, where feasible, randomized interventions (e.g., therapeutic vaccines or prophylactic measures) to more definitively establish causality and quantify benefits [82,83].

## 4. Discussion

This integrative review highlights that HPV exerts multifaceted effects throughout the reproductive lifespan. In men, HPV drives anogenital warts and oropharyngeal infections, and seminal HPV infection is also consistently linked to impaired sperm motility, altered morphology, and increased DNA fragmentation, thereby contributing to idiopathic infertility when compared to negative controls [4,13,19,84]. In women, persistent high-risk HPV not only drives cervical dysplasia but may also disrupt endometrial integrity and the cervical microenvironment, adversely affecting both natural conception and assisted reproductive technology outcomes [19,22].

During pregnancy, maternal HPV viral load, especially during the first trimester, emerges as a potent predictor of vertical transmission, with high DNA titers increasing transmission odds by more than sixfold [25]. Additionally, vaginal delivery, especially when membranes are ruptured for extended periods, is associated with elevated risks of both PROM and preterm birth, as well as low birth weight [9,39].

HPV can also be vertically transmitted (non-sexual route), contributing to neonatal respiratory papillomatosis and infant viral persistence, especially when maternal viral load is high.

Preventive measures, notably widespread nine-valent HPV vaccination coupled with extended-interval, HPV-based screening, are projected to reduce high-grade precancerous lesions by up to 80%, decrease the need for excisional procedures by over 75%, and consequently avert a substantial proportion of preterm deliveries linked to such treatments [85,86].

For oncological management in young women, fertility-sparing surgery (conization and radical trachelectomy) demonstrates low recurrence rates (<7%) while achieving pregnancy rates of up to 67% and live birth rates approaching 68% in properly selected cases [87,88,89].

**Figure 2:**
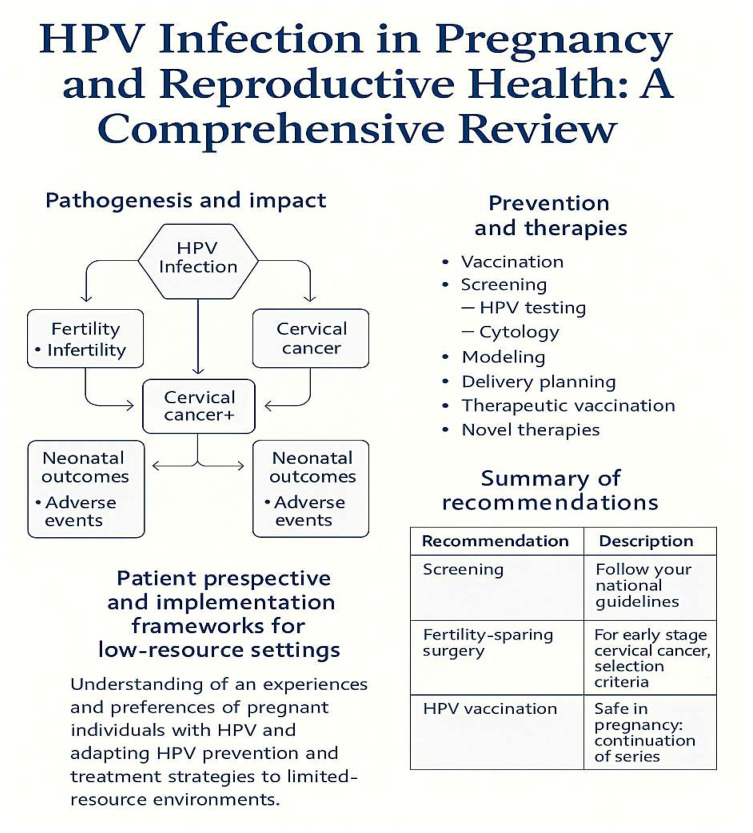
Schematic overview of HPV’s impacts and interventions across the reproductive lifespan. **Left**: Pathogenesis and outcomes (fertility, oncogenesis, neonatal). **Right**: Preventive and therapeutic interventions (vaccination, screening, delivery planning, novel therapies). **Bottom**: Patient perspective and implementation frameworks.

Emerging data on the cervical microbiome and molecular markers (e.g., p16/Ki-67, BCL-2, specific miRNAs) suggest additional pathways through which HPV may influence implantation and pregnancy maintenance, underscoring the need for future studies that integrate virological, immunological, and metabolomic analyses [4] (Figure 2).

### 4.1. Limitations of the Current Evidence

- Study Design Heterogeneity: Variability in populations, HPV genotypes, and clinical protocols limits generalizability.
- Population Bias: Many studies are based on selected or high-risk populations, reducing generalizability to broader reproductive cohorts.
- Scarcity of High-Quality RCTs: Several key reproductive endpoints lack support from adequately powered randomized controlled trials, limiting causal inference.
- Methodological Differences: Diverse assays for HPV detection, viral quantification, and outcome definitions complicate direct comparisons.
- Insufficient Long-term Data: Longitudinal evidence on the durability of outcomes post-intervention and long-term offspring health remains limited.
- Residual Confounding: Uncontrolled variables such as co-infections, lifestyle, and genetic predisposition may bias observed associations.

### 4.2. Patient Perspectives

Qualitative studies reveal that individuals value clear information on fertility implications and prefer minimally invasive treatments when cancer risk permits. Common concerns include:

- Reproductive Planning: Desire for reassurance on future fertility and timing of childbearing.
- Vaccine Hesitancy: Fears about vaccine safety during pregnancy
- Psychosocial Impact: Mitigating anxiety related to cancer diagnosis and fertility loss.

It could be useful to incorporate patient-reported outcomes (e.g., Fertility Quality of Life [FertiQoL] scores) in future trials to capture these dimensions.

### 4.3. Global Health Implementation

Low-Resource Settings:

- Vaccine Delivery: School-based, single-dose strategies can achieve >80% coverage.
- Screen-and-Treat Approaches: Visual inspection with acetic acid (VIA) plus immediate cryotherapy for HPV-positive or VIA-positive lesions where cytology/pathology is unavailable.
- Task-Shifting: Training mid-level providers to perform cryotherapy and simple trachelectomy expands access to fertility-sparing care.
- Community Engagement: Partnering with local leaders to address cultural barriers and misinformation enhances uptake.

### 4.4. Research Gaps and Future Directions

While substantial progress has been made in mapping the impact of HPV on reproductive health, critical gaps remain:

1. Mechanisms of Endometrial Tropism: The cell-surface receptors and intracellular pathways facilitating HPV entry into endometrial and fallopian tube epithelium are undefined. Organoid models and single-cell transcriptomics could elucidate how viral capsid proteins interact with host glycoproteins in these tissues.
2. Male Tract Histopathology: Detailed studies of HPV tropism in seminal vesicles, prostate, and epididymis and its impact on sperm integrity and reservoir function.
3. Long-Term Neonatal Outcomes: Outside of juvenile-onset RRP, the potential for HPV to influence neurodevelopmental trajectories, immune system maturation, or oncogenic risk in exposed neonates remains unstudied. Large, prospective birth cohorts with serial virologic and developmental assessments are urgently needed.
4. Vaccine Schedule Optimization: Emerging evidence suggests that extending the inter-dose interval may enhance immunogenicity, but randomized trials in immunocompromised and older adult populations are lacking.
5. Residual confounding from smoking and co-infections may bias observational estimates.
6. Tertiary-center samples limit generalizability to broader populations.
7. Implementation Science: Strategies to overcome vaccine hesitancy, expand access to FSS, and reduce regional disparities in screening and vaccination uptake.
8. Therapeutic Trials: Phase III (VGX-3100) and Phase II (GX-188E) studies of therapeutic vaccines and novel antivirals show early efficacy, and emerging personalized immunogenomic profiling promises more tailored interventions. Unfortunately, no formal human studies have evaluated the impact of therapeutic HPV vaccines or adoptive T-cell therapies on ovarian reserve, spermatogenesis, or early embryogenesis. Reproductive-toxicology assays and dedicated fertility endpoints should be integrated into future trials.
9. Mechanistic Insights: Translational research on HPV’s interaction with the cervical microbiome and host immunometabolic milieu with a multiomics approach.
10. Personalized Risk Profiling: Integration of clinical, molecular, and imaging data to develop individualized prognostic models and guide tailored interventions. In this regard, recent advances in machine learning risk stratification may complement HPV-based prognostic tools and enable more personalized screening intervals (Table 2) [90].

## 5. Conclusions

By synthesizing epidemiological, mechanistic, and clinical intervention evidence, this review underscores the profound impact of HPV on fertility, pregnancy outcomes, and survivorship among reproductive-aged individuals.

Key recommendations are:

- Integrated Prevention: Achieve high coverage with nine-valent vaccination and implement HPV-based screening at 5-year intervals to minimize CIN and the downstream need for excisional treatments.
- Delivery Planning: Personalize mode-of-delivery decisions, favoring vaginal birth with intact membranes when viral load is low, and balance obstetric safety with minimized vertical transmission risk.
- Risk Stratification: Employ validated nomograms and mid-trimester cervical length monitoring for women post-conization to guide prophylactic interventions (cerclage, progesterone).
- Fertility Preservation: Offer conservative surgical approaches (conization, radical trachelectomy) to eligible young women with early-stage cervical cancer to maintain reproductive potential.
- Comprehensive Surveillance: Establish long-term follow-up protocols for both mothers and offspring to detect late sequelae, including respiratory papillomatosis and growth or neurodevelopmental anomalies.

A multidisciplinary approach (gynecologic oncology, reproductive medicine, and maternal–fetal care) is essential to mitigate HPV’s diverse reproductive harms and optimize both oncologic and fertility outcomes.

## Figures and Tables

**Table 1:** Selected effect estimates for obstetric and neonatal outcomes.

*Outcome*	Effect Estimate (95% CI)	Study Design	GRADE Quality	References
*Risk of preterm premature rupture of membranes (PROM) with vaginal HPV infection*	OR 1.8 (1.3–2.4)	Cohort study	Moderate ^1^	Wu D et al., meta-analysis on PROM and preterm delivery (2021) [8]
*Preterm birth (<37 weeks)*	RR 1.5 (1.1–2.0)	Meta-analysis	Moderate ^2^	Wu D et al., meta-analysis on PROM and preterm delivery (2021) [8]
*HPV persistence in the infant at 12 months*	15% (95% CI 10–20%)	Prospective cohort study	Low ^3^	Trottier H et al., HERITAGE study on vertical transmission (2016) [26]

Notes on GRADE quality: ^1^ Cohort studies with moderate risk of bias and consistent findings. ^2^ Meta-analysis with moderate heterogeneity; outcome definitions largely comparable. ^3^ Small prospective studies with loss to follow-up, leading to imprecision. Background color has been added in order to improve readability.

**Table 2:** Synopsis of key recommendations.

*Domain*	Recommendation	Details	Strength of Evidence
*Primary Prevention*	Nine-valent HPV vaccination	Administer at ages 9–14 (2 doses), catch-up to 26 years (3 doses); continue series if pregnancy occurs	High (Phase III RCTs of prophylactic vaccines)
*Screening*	Primary HPV testing	Every 5 years from age 25; co-testing (HPV + cytology) every 5 years in vaccinated cohorts	High (Randomized screening trials and modeling studies)
*Delivery Planning*	Mode of delivery individualized	Vaginal delivery with intact membranes preferred unless high viral load or obstetric indication	Moderate (Cohort and meta-analytic evidence)
*Post-Conization Surveillance*	Risk-stratified cervical length measurement	Transvaginal measurement at 16–20 weeks; consider cerclage if <25 mm and high nomogram risk	Moderate (Validated nomogram in retrospective cohort)
*Fertility-Sparing Surgery*	Conization/simple trachelectomy	For ≤2 cm, no LVSI, negative nodes; radical trachelectomy for 1–2 cm with node assessment	Moderate (Systematic reviews and observational cohorts)
*Therapeutics*	Therapeutic vaccine trials	VGX-3100 or equivalent in Phase III with fertility & safety endpoints; enrolment encouraged	Low (Phase II trials; limited reproductive-safety data)

## Data Availability

Data are available upon reasonable request to the corresponding author.

## Appendix A

**Table A1:** PRISMA study.

Stage	Number of Records
Records identified (PubMed, Embase, Scopus)	3635
Records after deduplication	2109
Records screened	2109
Full-text articles assessed	73
Studies included in review	37
